# Forgone Health Care for Non–COVID-19–Related Needs Among Medicare Beneficiaries During the COVID-19 Pandemic, Summer 2020–Winter 2021

**DOI:** 10.5888/pcd19.220110

**Published:** 2022-10-13

**Authors:** Kara Tsuzaki, Deborah Taira

**Affiliations:** 1 The Daniel K. Inouye College of Pharmacy, Honolulu, Hawaii; 2 Now with Cynosure Consulting, LLC, Seattle, Washington

## Abstract

**Introduction:**

Forgone health care, defined as not using health care despite perceiving a need for it, is associated with poor health outcomes, especially among people with chronic conditions. The objective of our study was to examine how the pandemic affected forgone health care during 3 stages of the pandemic.

**Methods:**

We used the Medicare Current Beneficiary Survey COVID-19 Rapid Response Questionnaire administered in summer 2020, fall 2020, and winter 2021 to examine sociodemographic characteristics, chronic diseases, COVID-19 vaccination status, and telehealth availability in relation to beneficiary reports of forgone health care.

**Results:**

Of the 3 periods studied, the overall rate of forgone health care was highest in summer 2020 (20.8%), followed by fall 2020 (7.8%) and winter 2021 (6.5%). COVID-19 vaccination status, age, sex, race and ethnicity, US region, availability of primary care telehealth appointments, and chronic conditions (heart disease, arthritis, depression, osteoporosis or a broken hip, and diabetes or high blood glucose) were significantly related to forgone care.

**Conclusion:**

High rates of forgone care among Medicare participants varied over time and were significantly related to beneficiary characteristics. Our findings highlight the need for health care reform and changes in policy to address the issue of access to care for people with chronic conditions during a pandemic or other public health emergency.

**Summary**

**What is already known on this topic?**

The pandemic has affected access to health care even for conditions unrelated to COVID-19.

**What is added by this report?**

This study identified factors related to forgone care during the COVID-19 pandemic, including COVID-19 vaccination status, age, sex, race and ethnicity, US region, availability of primary care telehealth appointments, and chronic conditions (heart disease, arthritis, depression, osteoporosis or a broken hip, and diabetes or high blood glucose).

**What are the implications for public health practice?**

Our findings highlight the need for health care reform and changes in policy to address the issue of access to care for people with chronic conditions during a pandemic or other public health emergency.

## Introduction

The COVID-19 pandemic has led to widespread changes in the US health care system (1). Despite increases in the number of hospitalizations and related care for COVID-19 infections, health care use overall declined because people stopped accessing care for non–COVID-19–related conditions (2). Even with increased availability of telehealth options, forgone health care, defined as not using health care despite perceiving a need for it, increased during the pandemic (3–5).

People aged 65 years or older with comorbidities such as heart disease, lung disease, and diabetes are at higher risk than younger populations for severe health outcomes from COVID-19 (6). Additionally, people with chronic conditions may be more likely than those without to forgo care because of a lack of coordinated care between practitioners, hospitals, and clinics (7).

The problem of forgone health care encompasses multiple factors such as government and provider policy, perceived risk, and financial costs (8–10). The COVID-19 pandemic has directly affected all 3 factors. At the start of the pandemic, many states initiated stay-at-home orders that remained in place for months; elective surgeries were canceled, regular checkups were delivered via telehealth, clinics were voluntarily closed, and people elected to forgo needed care. Finally, the pandemic had vast financial effects: an economic downturn was directly linked to the decline in use of health care services (4).

Failure to make use of health care services has been associated with poor health outcomes (7). Previous studies identified disparities in health care access associated with sociodemographic factors such as sex, race and ethnicity, and income (11,12). Research has begun to identify similar predictors of forgone care and barriers to care during the COVID-19 pandemic (13–16). The level of forgone care was expected to be highest in the first stages of the pandemic because of the perceived risk of exposure to COVID-19 as well as operational and procedural barriers such as clinic closures and drug shortages. Our objective was to examine how COVID-19 affected forgone care during 3 stages of the pandemic — summer 2020, fall 2020, and winter 2021.

## Methods

We analyzed data from the Medicare Current Beneficiary Survey (MCBS) COVID-19 supplemental public use files for summer 2020 (17), fall 2020 (18), and winter 2021 (19). MCBS is a continuous and longitudinal survey that provides a representative national sample of the Medicare population. Medicare beneficiaries were contacted via telephone in June and July 2020 for the summer survey, October and November 2020 for the fall survey, and February, March, and April 2021 for the winter survey. The sample consisted of Medicare beneficiaries aged 65 years or older and people younger than 65 years with a Medicare-qualifying disability (eg, end-stage renal disease); 11,114 beneficiaries were interviewed for the summer survey, 9,686 for the fall survey, and 11,107 for the winter survey. All participants were continuously enrolled in Medicare and living in the community. Weighting was adjusted according to preliminary weights, eligibility, and completion of the survey. Because MCBS consists of de-identified, publicly available data, institutional review board approval was not sought for this study.

The primary dependent variable was forgone health care because of the pandemic. This information was self-reported in response to the question “Since [reference date], did you need medical care for something other than coronavirus, but not get it because of the coronavirus pandemic?” Forgone care included the need for urgent care, surgery, diagnostic tests, regular checkups, treatment of previous conditions, prescription drugs, and dental, vision, and hearing care. Information about the availability of telemedicine was obtained by asking, “Does your usual provider offer telephone or video appointments, so that you don’t need to physically visit their office or facility?” For information on vaccination status during winter 2021, beneficiaries were asked, “Since the [date of COVID-19 vaccine available] have you had a coronavirus vaccination?”

Sociodemographic characteristics assessed were age (<65, 65–74, or ≥75 y), sex (male or female), race and ethnicity (non-Hispanic Black, Hispanic, non-Hispanic White, or Other [any person that did not identify as non-Hispanic Black, Hispanic, or non-Hispanic White]), language spoken at home (only English spoken at home or other language besides English spoken at home), whether the respondent resided in a metropolitan (>50,000 people) or a nonmetropolitan area (≤50,000 people), US region of residence (Northeast, Midwest, South, or West), and annual household income (<$25,000 or ≥$25,000 per year).

Data on chronic conditions were self-reported by the beneficiary. For diabetes or high blood glucose, beneficiaries were asked, “Has a doctor or other health professional ever told [you] that [you/he/she] had any type of diabetes, including: sugar diabetes, high blood sugar, [borderline diabetes, pre-diabetes, or pregnancy-related diabetes/borderline diabetes, or pre-diabetes]?” The survey then asked about the beneficiary’s overall health and chronic conditions. We chose chronic conditions for this study on the basis of preliminary prevalence estimates of comorbidities associated with COVID-19 in the US as well as conditions that may present a physical obstacle to accessing care (20).

We weighted all frequencies to appropriately represent the national population. We used the χ^2^ test to conduct a cross-tabulation analysis of differences between each demographic characteristic and forgone care. *P* ≤ .05 indicates significance. We examined forgone care in relation to sociodemographic factors, vaccination status, telehealth availability, and our 5 selected chronic conditions. We used RStudio version 4.1.0 (RStudio Team) and Stata version 17 (StataCorp LLC) to perform all statistical analyses.

## Results

Overall, survey response rates were 78.9% for summer 2020, 72.6% for fall 2020, and 79.6% for winter 2021. The response rate for the primary variable of forgone care was 99.98% for all 3 surveys. The percentage of beneficiaries with forgone care was highest in summer 2020 (20.8%), followed by fall 2020 (7.8%) and winter 2021 (6.5%).

Demographic characteristics were similar in all 3 periods (Table 1). By age, the largest group of survey respondents was aged 65–74 years. Most were female (range, 54.6%–54.9%), approximately 75% were non-Hispanic White, almost 90% spoke only English at home, and approximately 80% lived in metropolitan areas. By region, the largest group lived in the South (range, 38.1%–39.5%), and approximately two-thirds had an annual household income of $25,000 or more.

**Table 1:** Demographic Characteristics of Medicare Beneficiaries During 3 Periods of the COVID-19 Pandemic, Summer 2020, Fall 2020, and Winter 2021^a^

Characteristic	Summer (June–July) 2020, %	Fall (October–November) 2020, %	Winter (February–April) 2021, %
**Age, y**			
<65	14.6	20.5	14.6
65–74	53.3	47.9	51.5
≥75	32.1	31.6	33.9
**Sex**			
Male	45.1	45.1	45.4
Female	54.9	54.9	54.6
**Race and ethnicity**			
Black, non-Hispanic	9.9	9.7	9.6
Hispanic	8.0	8.4	8.4
Other^b^	6.4	6.3	6.2
White, non-Hispanic	75.7	75.7	75.8
**Language**			
Other language besides English spoken at home	11.0	11.4	11.1
Only English spoken at home	88.9	88.6	88.9
**Residence**			
Metropolitan	79.8	80.1	80.0
Nonmetropolitan	20.2	19.9	20.0
**US region**			
Northeast	17.9	17.9	17.5
Midwest	22.1	22.0	21.5
South	38.1	38.3	39.5
West	21.8	21.9	21.6
**Annual household income, $**			
<25,000	30.6	30.8	29.3
≥25,000	65.7	65.6	67.4

a Data source: Centers for Medicare & Medicaid Services, Medicare Current Beneficiary Survey COVID-19 supplements (17–19). Not all survey participants answered all questions; percentages may not sum to 100%.

b “Other” includes any person that did not identify as non-Hispanic Black, Hispanic, or non-Hispanic White.

In winter 2021, beneficiaries who were vaccinated for COVID-19 were significantly less likely than unvaccinated beneficiaries to forgo health care (6.5% vs 6.7%; *P* < .001).

Forgone care was significantly related to many demographic characteristics (Table 2). Beneficiaries aged younger than 65 years had the highest rates of forgone care, by age, in all 3 periods (21.7%, 9.5%, and 10.2%, respectively; *P* < .001). Other significant predictors were sex, race and ethnicity, US region, and annual household income. Women were more likely than men to forgo care in summer 2020 (21.6% vs 19.7%; *P* = .05). By race and ethnicity, non-Hispanic White beneficiaries (22.5%) were most likely and non-Hispanic Black beneficiaries (12.6%) were least likely to forgo care in summer 2020 (*P* = .002); however, we found no significant differences by race and ethnicity in fall 2020 or winter 2021. By US region, the Midwest was most likely (23.5%) and the South least likely (16.6%) in summer 2020 to forgo care (*P* = .001). In winter 2021, however, the West was most likely (8.2%) and the South least likely (5.3%) (*P* = .03). In summer 2020, an annual household income of $25,000 or more was associated with an increase in forgone care. We found no significant differences by income in fall 2020, but in winter 2021, an annual household income less than $25,000 was associated with more forgone care (7.0%) than an income of $25,000 or more (6.3%).

**Table 2:** Demographic Characteristics of Medicare Beneficiaries Who Reported Forgoing Non–COVID-19–Related Health Care During 3 Periods, Summer 2020, Fall 2020, and Winter 2021^a^

Characteristic	Summer 2020		Fall (October–November) 2020		Winter (February–April) 2021	
	%	*P* value^b^	%	*P* value^b^	%	*P* value^b^
**Reported forgone care due to COVID-19^c^**	20.8	—	7.8	—	6.5	—
**Age, y**						
<65	21.7	<.001	9.5	.003	10.2	<.001
65–74	22.3		8.1		6.6	
≥75	17.8		6.3		4.9	
**Sex**						
Male	19.7	.05	7.5	.57	5.8	.07
Female	21.6		8.0		7.1	
**Race and ethnicity**						
Black non-Hispanic	12.6	.002	7.0	.68	6.8	.30
Hispanic	16.3		8.5		8.0	
Other^d^	18.4		7.6		6.9	
White non-Hispanic	22.5		7.8		6.3	
**Language**						
Other language besides English spoken at Home	15.6	.11	7.3	.06	7.2	.22
Only English spoken at Home	21.4		7.9		6.5	
**Residence**						
Metropolitan	20.9	.81	7.8	.62	6.7	.06
Nonmetropolitan	20.1		7.9		5.8	
**US Region**						
Northeast	23.2	.001	8.4	.29	8.0	.03
Midwest	23.5		7.7		6.0	
South	16.6		6.9		5.3	
West	23.4		9.1		8.2	
**Annual household income, $**						
<25,000	15.5	<.001	7.2	.20	7.0	.008
≥25,000	23.6		8.2		6.3	

Abbreviation: — , does not apply.

a Data source: Centers for Medicare & Medicaid Services, Medicare Current Beneficiary Survey COVID-19 supplements (17–19).

b Determined by χ^2^ test.

c Question on survey was “Since [reference date], did you need medical care for something other than coronavirus, but not get it because of the coronavirus pandemic?”

d “Other” includes any person that did not identify as non-Hispanic Black, Hispanic, or non-Hispanic White.

The proportion of primary care providers (PCPs) that beneficiaries said offered telehealth was similar in summer 2020, fall 2020, and winter 2021 at 60.1%, 62.8%, and 63.4%, respectively. The highest proportion of forgone care related to telehealth access was reported in summer 2020: 22.8% of beneficiaries whose PCP offered telehealth reported forgoing care, while 18.1% whose PCPs did not offer telehealth reported forgoing care (Figure). In all 3 periods, beneficiaries whose PCP offered telehealth appointments were more likely to forgo care.

**Figure:**
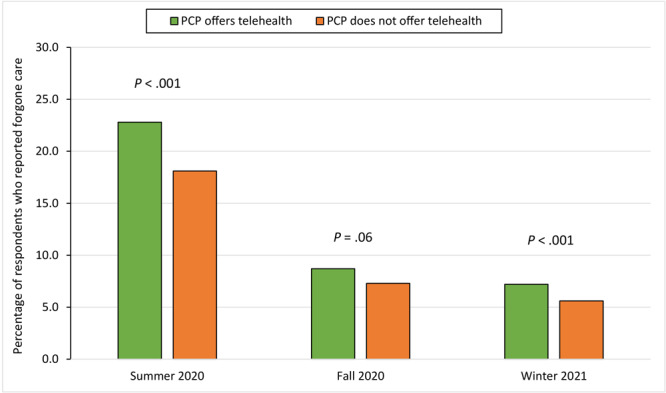
Percentage of Medicare beneficiaries who indicated forgoing health care, by whether primary care providers offered telehealth, summer 2020, fall 2020, and winter 2021. Availability of telemedicine was measured by asking Medicare beneficiaries, “Does your usual provider offer telephone or video appointments, so that you don’t need to physically visit their office or facility?” Abbreviation: PCP, primary care provider. Data source: Centers for Medicare & Medicaid Services (17–19).

**Table:** 

Period	Percentage of Medicare beneficiaries who reported forgoing health care		*P* value
	PCP offers telehealth	PCP does not offer telehealth	
Summer 2020	22.8	18.1	Less than .001
Fall 2020	8.7	7.3	.06
Winter 2021	7.2	5.6	Less than .001

Chronic conditions that were significantly associated with forgone care in all 3 periods were heart disease, arthritis, and depression (Table 3). For example, 22.4% (*P* = .01) of beneficiaries with heart disease reported forgoing care in summer 2020, 8.6% (*P* = .05) in fall 2020, and 7.6% (*P* = .01) in winter 2021. Additionally, osteoporosis or a broken hip was significantly associated with forgoing care in summer 2020 and winter 2021. Finally, diabetes was significantly associated with forgone care in fall 2020.

**Table 3:** Selected Chronic Conditions Among Medicare Beneficiaries Who Responded to a Survey on Forgone Health Care During 3 Periods, Summer 2020, Fall 2020, and Winter 2021^a^

Characteristic	Summer 2020		Fall 2020		Winter 2021	
	% of Respondents who reported forgoing care	*P* value^b^	% of Respondents who reported forgoing care	*P* value^b^	% of Respondents who reported forgoing care	*P* value^b^
Heart disease	22.4	.01	8.6	.05	7.6	.01
Diabetes or high blood glucose	22.0	.17	6.9	.001	7.3	.71
Arthritis	22.5	<.001	9.4	.001	6.2	.05
Osteoporosis or broken hip	24.2	<.001	9.4	.14	8.0	.04
Depression	24.8	.002	10.4	<.001	9.2	<.001

a Data source: Centers for Medicare & Medicaid Services, Medicare Current Beneficiary Survey COVID-19 supplements (17–19).

b Difference between percentage of people who self-reported having a chronic disease and percentage who did not; determined by χ^2^ test.

## Discussion

In this study of Medicare survey respondents at 3 time points during the COVID-19 pandemic, we found a significant relationship between high rates of beneficiaries forgoing health care and beneficiary characteristics. Age was significantly associated at all 3 time points. By age group, beneficiaries aged 65 years or younger were the most likely to forgo care. These beneficiaries have disabilities such as end-stage renal disease, amyotrophic lateral sclerosis (ALS), and other clinical conditions that severely diminish their ability to work and they qualify for Medicare through a rigorous, consistent review process. Previous studies showed that people in this group are likely to identify as a racial or ethnic minority, be of low socioeconomic status, report mental health conditions, have comorbidities, and have high health care expenditures (21).

Chronic conditions were also a strong predictor of forgone care, especially heart disease, arthritis, and depression. According to the Centers for Disease Control and Prevention, the risk of severe illness from COVID-19 increases with age and number of underlying medical conditions. Diseases such as chronic kidney disease (any stage), dementia and other neurologic conditions, and disabilities are linked to a high risk for severe illness from COVID-19 (22). These conditions may have limited the type of care and risk of accessing care. Mental health status is also associated with disparities in health care access (16,23,24) and may be related to gaps in Medicare coverage for mental health disorders (24).

In our study, women were significantly more likely than men to forgo care in summer 2020. This mirrors a national trend in which more women than men forgo care (25). The decision to forgo care might result from gender-related differences in thoughts about COVID-19, perceived and real risks of COVID-19, and economic concerns (25,26). Our study showed that higher income (≥$25,000 per year) predicted not accessing care in summer 2020, but the opposite was true in winter 2021. Socioeconomic disparities in access to care are not as extreme in the Medicare population as in the general US population. However, differences still exist, potentially from gaps in coverage, high out-of-pocket costs, and lack of knowledge about the availability of telehealth and the ability to use it (27).

Race and ethnicity significantly predicted forgoing care in summer 2020, with the highest rates among non-Hispanic White beneficiaries. Although racial and ethnic disparities exist in US health care, such disparities are smaller among Medicare beneficiaries in terms of insurance coverage, access to care, and self-reported health (28). Our study showed that race and ethnicity were not significant predictors of not accessing care in fall 2020 or winter 2021.

In comparisons of US regions, the South is commonly associated with low levels of access to quality care (28,29). However, in our study of Medicare beneficiaries, by US region, the South had the lowest percentage of beneficiaries who did not have access needed health care in summer 2020 and winter 2021. It is possible the South has access-to-care issues unrelated to COVID-19 or access to care.

Overall, our findings may have been confounded by socioeconomic status, education, and overall feelings about COVID-19. Studies have associated race and ethnicity with perceptions of COVID-19 (10,12,26). Racial and ethnic minority populations were less likely than the White population to be vaccinated and more likely to disagree that COVID-19 is more severe than influenza during the early phases of the COVID-19 pandemic (26,30,31).

We found that in winter 2021, COVID-19–vaccinated beneficiaries were less likely than unvaccinated beneficiaries to forgo care. The availability and receipt of vaccines may have affected the overall reduction in forgone care from fall 2020 to winter 2021. However, the difference in the percentage of beneficiaries who reported forgoing care was small: 6.7% among unvaccinated beneficiaries and 6.5% among vaccinated beneficiaries. Potential confounders might include the passage of time or a sense of safety resulting from the availability and receipt of the COVID-19 vaccination.

Although the overall number of PCPs who reportedly offered telehealth services remained consistent over time, access to care may have been affected by logistical barriers. With the announcement of clinic closures and surgery cancellations, physicians may not have been prepared to offer telehealth at the scale needed or may have been unable to offer in-person visits. This factor may have affected the initial reduction in forgoing care from summer 2020 to fall 2020 as health care providers became better equipped to handle telehealth appointments.

### Limitations

Our study has several limitations. First, all data were self-reported, which may have introduced recall bias, sampling bias, social desirability bias, and varying levels of introspection. The period of forgone care could have been recalled incorrectly. The survey also asked beneficiaries to self-report chronic diseases; we had no information on whether they were being treated for them. Additionally, although data were reported by US region, COVID-19 guidelines and mandates varied by state. Each supplemental survey covered a short period (2 or 3 months), which may not have allowed certain mandates or perceived risks to be implemented and understood. Although the MCBS sampling frame allows for estimates of national averages, it does not allow for an in-depth analysis of racial or ethnic minority groups, undocumented workers, or other groups of people who are socially and economically marginalized. Because of small sample sizes, analyses of non-Hispanic Black and Hispanic beneficiaries were especially susceptible to sampling errors (32). We could not identify additional racial and ethnic groups and included these under the general term “other.” These groups are affected by small sample sizes and also by the limitation of the survey being offered only in English and Spanish. Furthermore, because MCBS is conducted by the US government, people who have experienced historical trauma may not be inclined to participate.

### Conclusion

Our study identified associations between various factors and access to health care among Medicare beneficiaries during the COVID-19 pandemic. Our results are corroborated by Park and Stimpson in regard to the significance found for age, chronic conditions, sex, income, race and ethnicity, and region (16). Their study highlighted the effect of physician-driven factors, such as availability of telehealth consultations, and mental health status on beneficiaries’ decision to forgo health care. Our study includes an analysis of vaccination status and emphasizes multiple chronic conditions and beneficiary characteristics in relation to nonuse of care. It highlights the need for further research and policy change for Medicare beneficiaries in the US, especially populations with multiple chronic health conditions and low socioeconomic status. Additionally, future research is needed to fully understand the extent of forgone care among communities that are socially and economically marginalized.

Forgone or postponed care can have long-term health consequences. The lack of preventive care, including screenings and vaccinations, can lead to delayed treatment or not receiving proper care. Examples of interventions include the Coronavirus Aid, Relief, and Economic Security Act (33), which provided financial support to patients and medical providers, thereby decreasing barriers to care. Further research pertaining to access to care will continue to change with the introduction of booster shots, relaxation of mask and social distancing mandates, and perceived risks and attitudes toward COVID-19.
